# A Cure for Snake Bite

**Published:** 1880-08

**Authors:** 


A Cure for Snake Bite.—Mr. Tremlett, Consul for Saigon, furnishes, according to the *Medical Press and Circular*, July 7th, 1880, some further notes respecting *hwang-nao*, which is said to cure the bite of the most venemous serpents, and has been successfully employed in curing cancer, and principally leprosy, in the treatment of which it has never given other than satisfactory results. The native physicians, Mr. Tremlett tells us, distinguish thirty-six kinds of leprosy, the most common attacking the feet and hands ; it is considered to be hereditary, and is usually contracted by children at the age of puberty. After some generations, it has been noticed to confine itself to either the male or the female members of the family; the disease is considered contagious, which only would account for the number of lepers in Tonking.

